# Targeting the C3 signaling axis of the complement system: immune microenvironment regulation and emerging therapeutic strategies for glioblastoma

**DOI:** 10.3389/fimmu.2026.1806462

**Published:** 2026-04-10

**Authors:** Jianhuang Huang, Qixiu Wang, Jianning Chen

**Affiliations:** 1 Department of Neurosurgery, Affiliated Hospital of Putian University, Putian, Fujian, China; 2 Department of Cerebral Diseases Rehabilitation III, Affiliated Hospital of Liaoning University of Traditional Chinese Medicine, Shenyang, Liaoning, China

**Keywords:** biomarkers, C3a receptor, complement C3, glioblastoma, hypoxia, targeted therapy, tumor microenvironment, tumor-associated macrophages

## Abstract

Glioblastoma (GBM) is the most malignant primary brain tumor, with treatment resistance tightly linked to the intricate regulation of the tumor immune microenvironment (TME). In recent years, the complement system—especially its central component C3 and its cleavage products C3a/C3aR signaling—has drawn increasing attention for its roles in GBM initiation and progression. This review systematically delineates the expression patterns of C3 in GBM, its regulatory mechanisms, and its connections to key biological processes such as hypoxia, immunosuppression, and angiogenesis. We provide a comprehensive analysis of the potential of targeting C3/C3aR signaling as a novel GBM therapy, including the application of small-molecule antagonists, synergistic effects with radiotherapy, and the prospects for biomarker development based on liquid biopsy. All therapeutic claims are based predominantly on preclinical evidence; clinical translation remains to be validated. By integrating the latest findings, this work aims to offer new perspectives and theoretical support for understanding GBM immune evasion and for the development of precise immunotherapies.

## 1 Introduction

Glioblastoma (GBM) is the most aggressive primary brain tumor in adults, with notoriously poor prognosis—the five-year survival rate remains below 7% (1). This grim outlook largely stems from pronounced tumor heterogeneity, intrinsic resistance to radiotherapy and chemotherapy, and the complex tumor microenvironment (TME) (2). GBM’s TME is a multifaceted network of malignant cells, normal brain cells, and immune cells that communicate via direct contact or paracrine signals (e.g., cytokines, neurotransmitters, and extracellular vesicles) to promote tumor proliferation, angiogenesis, immunosuppression, and therapy resistance (1). Among the cellular constituents of the TME, immunosuppressive cells—most notably tumor-associated macrophages (TAMs) and myeloid-derived suppressor cells (MDSCs)—dominate and are pivotal drivers of immune escape and tumor progression (3, 4). Evidence indicates that GBM’s immune microenvironment is marked by monocyte/macrophage enrichment and immunosuppression, with M2-like/immunosuppressive TAMs closely linked to poor patient prognosis (5, 6). These M2-like TAMs not only secrete pro-tumor factors such as vascular endothelial growth factor (VEGF) and transforming growth factor-β1 (TGF-β1) to promote tumor growth, angiogenesis, and epithelial–mesenchymal transition (EMT), but also suppress effector immune cells such as CD8+ T cells, thus creating an immunosuppressive niche favorable to tumor progression (7–9).

Traditionally, the complement system has been viewed as a core effector arm of innate immunity, primarily clearing pathogens and abnormal cells. However, recent work reveals a paradoxical role for complement in the tumor microenvironment, acting as a double-edged sword: on one hand, complement activation can mediate antibody-dependent cellular cytotoxicity (ADCC) and phagocytosis to fight cancer; on the other hand, tumors can hijack complement activation products to foster chronic inflammation, angiogenesis, immunosuppression, and metastasis within the TME (10–13). C3, the linchpin of the complement cascade, yields C3a, which signals through its receptor C3aR and has emerged as a key regulator of the tumor microenvironment, particularly of myeloid cell function (14, 15). Throughout this review, C3AR1 denotes the gene encoding the C3a receptor, while C3aR refers to the corresponding protein; C3AR1 is used when citing transcriptomic data and C3aR when discussing protein function and signaling pathways. In GBM, C3 and C3aR are upregulated and associated with poor prognosis (14). Notably, critical GBM TME drivers such as hypoxia and TGF-β1 upregulate the C3/C3aR axis; hypoxia increases C3 and C3aR expression in GBM cells and stroma, while TGF-β1 (based on *in vitro* evidence from THP-1–derived macrophages (15)) robustly upregulates VEGF, C3, and C3aR in TAMs. This upregulated C3a/C3aR signaling promotes TAM polarization toward an M2-like/immunosuppressive phenotype, enhancing pro-angiogenic and immunosuppressive functions and forming a vicious hypoxia–TGF-β1–C3a/C3aR–immunosuppressive TAMs loop that aggravates GBM invasiveness and therapy resistance (14, 15). Therefore, a detailed dissection of C3/C3aR signaling in GBM’s TME and the exploration of pathway-targeted therapies to reverse immunosuppression and boost anti-tumor immunity hold significant theoretical and clinical promise.

## 2 Expression features and clinical significance of C3/C3aR signaling in GBM

### 2.1 High expression of C3 and C3aR in GBM tissue and cells

Several transcriptomic analyses support the high and GBM-specific expression of complement C3 and its receptor C3aR (gene: C3AR1). Analyses of bulk RNA-seq, single-cell RNA-seq, and spatial transcriptomics data show that C3 and C3AR1 are upregulated in IDH-wild-type GBM and IDH-mutant grade 4 astrocytoma relative to lower-grade gliomas or other brain tumors (14). Additional work confirms that C3aR is markedly upregulated in grade 4 diffuse gliomas, particularly IDH-wild-type GBM and IDH-mutant grade 4 astrocytoma, with much lower expression in other brain tumors (15). Together, these data establish a GBM-wide activation of the C3/C3aR axis in high-grade gliomas. At the protein level, immunohistochemistry reveals predominant C3aR expression within the tumor parenchyma, especially on TAMs co-expressing CD68 and CD163; these TAMs also co-express VEGF, a pro-angiogenic factor (15). The colocalization suggests C3aR signaling may participate in TAM functional regulation and the acquisition of a pro-angiogenic phenotype. Importantly, recent single-cell transcriptomic studies have revealed that GBM-associated myeloid populations exist along a continuous spectrum rather than discrete M1/M2 categories; C3aR^+^ TAMs display immunosuppressive and pro-angiogenic features that overlap with—but are not identical to—classical M2 markers (14). C3 protein itself is detected in GBM tissue and shows substantial spatial heterogeneity. In a GBM mouse model, C3 is specifically enriched in hypoxic tumor regions (14). *In vitro*, hypoxia upregulates C3 and C3aR in multiple GBM cell lines and stromal cells in an oxygen-dependent manner (14). Collectively, C3 expression in GBM is tightly linked to intratumoral hypoxia, suggesting hypoxia as a key upstream driver of local complement activation. Across transcriptional to proteomic levels, and across whole tumor to specific cell subpopulations and spatial regions, the high and GBM-specific expression pattern of C3/C3aR is corroborated by multi-omics data, underpinning further exploration of their function and therapeutic potential.

### 2.2 C3/C3aR expression and patient prognosis

Clinical analyses show that high C3 and C3aR expression correlate with more invasive disease and shorter overall survival, underscoring their potential as adverse prognostic biomarkers. In human gliomas, C3 and its receptor C3AR1 associate with aggressive disease and shorter survival (14), with a stronger signal in higher-grade gliomas, suggesting that the activation level of the C3/C3aR axis may be a key driver of malignant progression. In addition to prognostication, pathway activation may influence treatment responses. Tumor recurrence and radiotherapy resistance are closely linked to hypoxia (14). Given that C3 expression is linked to hypoxic TME, the degree of C3/C3aR activation is likely tied to recurrence risk and radiation resistance. Preclinical evidence supports this: in a syngeneic murine tumor model (*in vivo*), C3aR antagonist SB290157, used alone or with radiotherapy, prolongs survival (14). This therapeutic effect partly results from reduced M2-like/immunosuppressive macrophages, which are associated with immunosuppression and tumor progression. Thus, assessing C3/C3aR expression may identify patient subgroups at high risk of recurrence and potential radiotherapy resistance, offering a molecular basis for risk stratification. Targeting this axis to reverse immunosuppression and improve radiotherapy sensitivity holds promise for improved outcomes. In sum, the C3/C3aR axis links hypoxia, immunoregulation, and therapy resistance and emerges as a potent prognostic biomarker and therapeutic target in GBM.

## 3 Upstream regulatory mechanisms of GBM microenvironment on C3/C3aR signaling

### 3.1 Hypoxia as a central driver

Hypoxia is a hallmark GBM microenvironment feature, directly associated with radiotherapy resistance and recurrence and driving malignant progression (16). Multiple studies show hypoxia triggers a cascade of immunosuppressive processes and malignant cellular responses that promote progression and poor prognosis (16). In GBM, hypoxia is not only ubiquitous but also a principal force shaping an immunosuppressive TME (17). Spatial transcriptomics and single-cell sequencing reveal nuanced hypoxic effects: in primary IDH-wild-type GBM, tumor subtypes exhibit distinct hypoxia states, with mesenchymal-like (MES-like) tumors experiencing more severe hypoxia (18). GBM MES-like subtypes harbor intrinsic mesenchymal transcriptional programs governed partly by tumor-cell-intrinsic regulatory states, independent of hypoxia (19, 20). Hypoxia may further amplify these pre-existing programs or partially induce mesenchymal features in other subtypes; the relative contributions of intrinsic and extrinsic factors therefore warrant careful distinction. Hypoxia drives not only mesenchymal transition in tumor cells but also modulates intercellular communication to stimulate tumor proliferation and invasion (18). Importantly, hypoxia fosters macrophage polarization and upregulates inhibitory intercellular interactions, markedly suppressing immune cell activity within the TME (18). Single-cell spatial analyses corroborate this: the hypoxia-linked upregulation of CD73 in tumor cells and spatial proximity of CD39-expressing microglia correlate with poor prognosis, fostering an adenosine-rich immunosuppressive milieu (21). Moreover, hypoxia can remodel the tumor immune microenvironment via epitranscriptomic mechanisms; hypoxia-inducible RNA demethylase ALKBH5 stabilizes long noncoding RNA NEAT1 and promotes CXCL8/IL-8 secretion, recruiting TAMs and dampening immunity (22). Together, these studies establish a causal axis of hypoxia–immunosuppression–tumor malignancy, highlighting hypoxia as a central driver of GBM’s pathogenesis.

### 3.2 Regulation by the key cytokine TGF-β1

Transforming growth factor-β1 (TGF-β1) is a pervasive cytokine in the GBM TME that orchestrates immunosuppression and pro-tumor phenotypes. Evidence indicates that TGF-β1 acts in concert with hypoxia and other microenvironmental cues to sculpt an immunosuppressive milieu. For example, high FBP1 expression in GBM associates with poor prognosis and correlates positively with multiple immunosuppressive factors, including TGF-β1 (23), suggesting TGF-β1 as a pivotal node in immunosuppressive networks. *In vitro* studies using THP-1–derived macrophages demonstrate that TGF-β1 can upregulate C3 and C3aR expression and promote a pro-angiogenic phenotype (15); direct transcriptomic validation of this association in primary patient-derived TAMs is, however, currently lacking and warrants future investigation. TGF-β1 signaling is widely implicated in shaping GBM’s immune milieu. TGF-β1 likely promotes TAM pro-tumor polarization via Smad and other downstream pathways. For instance, tumor-associated foam cells (TAFs), a macrophage subpopulation rich in lipid droplets, display pro-tumor features in GBM associated with hypoxia, mesenchymal transition, and angiogenesis (24). Additionally, MDSCs—an important component of GBM’s immunosuppressive milieu—are driven by tumor-derived exosomal miR-1246; hypoxia upregulates POU5F1 and hnRNPA1 to enhance miR-1246 transcription and packaging into exosomes (25). Overall, TGF-β1 stands as a key immunomodulatory factor in GBM, though its direct regulatory role on the C3/C3aR axis in primary GBM TAMs requires further experimental verification. TGF-β1 likely cooperates with other hypoxia-induced signals (e.g., HIFs, exosomal miRNAs) to regulate downstream pathways and consolidate TAM pro-angiogenic and immunosuppressive functions, thereby sustaining tumor progression and immune evasion.

### 3.3 Involvement of complement activation pathways

Available evidence suggests that the GBM microenvironment harbors locally active complement components, with complement activation potentially linked to immune evasion and tumor progression (14, 15); however, the precise pathways and cellular sources of complement activation in GBM remain incompletely characterized and warrant further investigation (10, 11). In GBM, tumor and stromal cells actively participate in shaping local immune regulation. For instance, purinergic signaling represents another hypoxia-driven immunosuppressive mechanism: tumor cells predominantly express CD73, while microglia are a major source of CD39; their spatial colocalization supports adenosine production and a locally immunosuppressive milieu (21). Importantly, the CD73/adenosine axis and the C3a/C3aR axis should be understood as parallel, independently operating hypoxia-driven immunosuppressive pathways that both contribute to the immunosuppressive TME in GBM. Current evidence does not support direct mechanistic cross-regulation between these two systems; rather, both are upregulated under hypoxic conditions and converge functionally to suppress anti-tumor immunity through distinct molecular mechanisms. Hypoxia can upregulate tumor cell CD73 (21) and promote adenosine accumulation (26). Likewise, generation and regulation of complement components may be co-controlled by tumor and stromal cells. In GBM, locally produced C3 can be cleaved into C3a and C3b by complement convertases activated through the classical, alternative, or lectin pathways; local inflammatory signals and immune complexes within the tumor microenvironment can trigger such activation. The specific predominant pathway of C3 activation in GBM requires further mechanistic study.

## 4 Downstream tumor-promoting effects of the C3a/C3aR axis

### 4.1 Induction of M2 polarization in tumor-associated macrophages

Within GBM’s immune milieu, the C3/C3aR axis—via C3a and C3aR—plays a central role in driving TAMs toward an M2-like/immunosuppressive phenotype. The classical M1/M2 dichotomy is, however, an oversimplification: single-cell transcriptomic studies demonstrate that GBM-associated TAMs exist along a continuous functional spectrum, and C3aR^+^ TAMs represent a subpopulation with immunosuppressive and pro-angiogenic features that partially overlap with M2 markers without constituting a homogeneous M2 population (14, 27). NFAT1 has been shown to upregulate C3 transcription in TAMs, increasing C3a secretion (28). Secreted C3a binds to C3aR on macrophages, activating the Ca2+ signaling axis and upregulating NFAT1 activity, which in turn promotes C3 transcription and C3a release, forming a positive feedback loop that sustains immunosuppressive polarization and supports tumor growth (28). This polarization phenomenon is not unique to GBM; in other cancers such as lung cancer, C1q and other complement components can induce TAM M2-like polarization (29). Once polarized to an immunosuppressive state, TAMs secrete immunosuppressive cytokines such as IL-10, which promotes tumor progression through downstream signaling (e.g., STAT3) in several cancers (30). In colorectal cancer, TAMs driven by Wnt5a sustain M2-like polarization via autocrine IL-10, further promoting growth and metastasis (31). In addition to cytokine secretion, immunosuppressive TAMs reprogram metabolism (e.g., arginine depletion) to suppress effector T cell function, establishing a profoundly immunosuppressive TME that facilitates immune escape (32). Thus, C3a/C3aR-driven immunosuppressive TAM polarization is a core mechanism underlying GBM’s immunosuppressive TME and tumor immune evasion.

### 4.2 Promotion of tumor angiogenesis

The C3a/C3aR axis not only modulates immune cell phenotypes but also intersects with another GBM hallmark—abnormal angiogenesis—forming an integrated “complement–immune cell–angiogenesis” regulatory network. Spatial co-localization of C3aR-positive TAMs with VEGF expression in GBM tissue suggests an association between C3a/C3aR signaling and pro-angiogenic TAM function (15); mechanistically, C3a/C3aR-driven immunosuppressive polarization is known to promote VEGF upregulation in TAMs (28), though direct causal evidence that C3aR activation alone is sufficient to induce VEGF transcription requires further functional validation. For example, in other cancers, tumor cells undergoing aerobic glycolysis produce lactate that drives TAM M2-like polarization and subsequent IL-6 secretion, promoting tumor aggressiveness and angiogenesis (33). In GBM, glioma stem cell (GSC)–derived exosomal miR-374b-3p can be internalized by macrophages and downregulate PTEN, inducing M2-like polarization, which then enhances endothelial cell migration and tubulogenesis in co-culture, promoting angiogenesis (34). Moreover, GBM cells upregulate KDELC2 to enhance mitochondrial ROS and ER stress, driving TAM proliferation and boosting pro-angiogenic factor expression, collectively promoting tumor vascularization (35). Hence, the C3a/C3aR axis fosters a microenvironment rich in immunosuppressive TAMs that indirectly but powerfully promotes aberrant neovascularization, linking complement activation with GBM’s vascular pathology.

### 4.3 Enhancement of tumor invasiveness and therapy resistance

**Figure 1:**
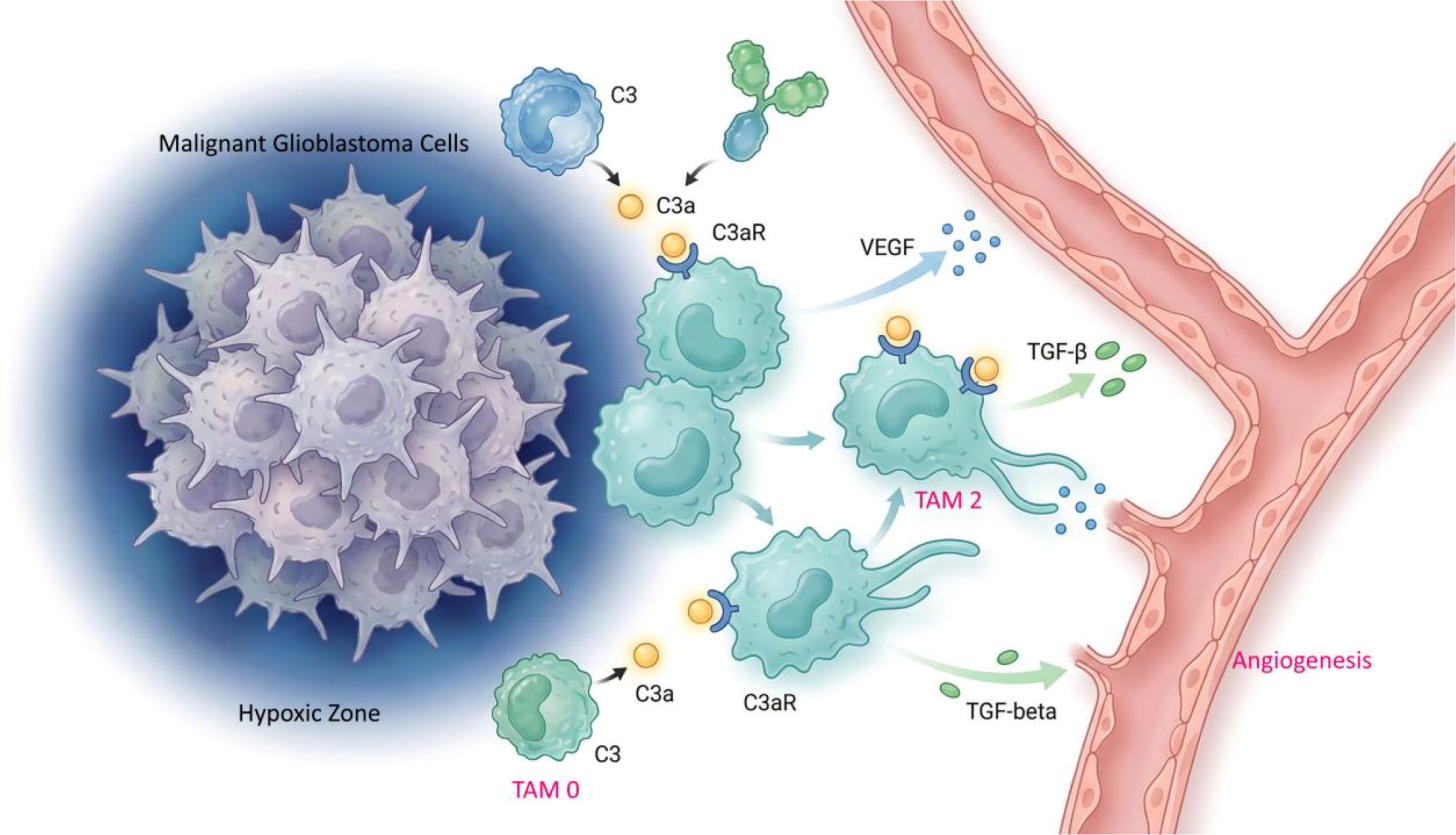
The hypoxia-driven C3/C3aR axis orchestrates an immunosuppressive and angiogenic microenvironment in glioblastoma. In the core region of glioblastoma (GBM), a severely hypoxic microenvironment synergizes with elevated TGF-β1 to drive marked upregulation of C3 expression and secretion by tumor cells and surrounding stroma. Locally produced C3 protein is subsequently activated through proteolytic cleavage by complement convertases—assembled via the classical, alternative, or lectin pathways in response to local inflammatory signals and immune complexes within the tumor microenvironment—generating the bioactive fragment C3a and the opsonin C3b. C3 expression (transcriptional upregulation driven by hypoxia and TGF-β1) and C3 activation (convertase-mediated proteolytic cleavage) are biochemically distinct steps; the predominant complement activation pathway in GBM requires further mechanistic investigation. Acting as a key signaling molecule, C3a binds specifically to the C3a receptor (C3aR) on tumor-associated macrophages (TAMs). C3a/C3aR engagement, through downstream Ca²^+^/NFAT1 signaling, promotes the polarization of TAMs toward the M2-like/immunosuppressive phenotype. Recent single-cell transcriptomic studies demonstrate that GBM-associated TAMs exist along a continuous functional spectrum; C3aR^+^ TAMs display immunosuppressive and pro-angiogenic features that overlap with—but are not identical to—classical M2 markers, and should not be equated with a discrete M2 population. These M2-like/immunosuppressive TAMs subsequently secrete abundant VEGF—spatial co-localization of C3aR^+^ TAMs with VEGF expression has been observed by immunohistochemistry, suggesting an association between C3a/C3aR signaling and pro-angiogenic TAM function; however, direct causal evidence that C3aR activation alone is sufficient to induce VEGF transcription requires further functional validation—to promote angiogenesis and IL-10 to suppress T cell function, thereby forming a self-reinforcing hypoxia–C3a–M2-like/immunosuppressive TAMs–angiogenesis pro-tumor loop.

The C3a/C3aR axis augments GBM invasiveness and resistance to conventional therapies by two routes: direct effects on tumor cells and indirect remodeling of the immune microenvironment. Directly, C3a/C3aR signaling may activate survival and proliferation pathways such as PI3K/Akt and MAPK in tumor cells. In colorectal cancer, FNDC4 activates Akt/STAT3 to promote epithelial–mesenchymal transition (EMT), migration, and invasion (36). In GBM, β2-microglobulin (B2M) interacts with PIP5K1A to activate the PI3K/AKT/mTOR axis, sustaining glioma stem cell (GSC) traits and promoting self-renewal and tumor growth (37). Indirectly, immunosuppressive TAMs recruited and polarized by C3a/C3aR create an immunosuppressive, pro-survival environment, a principal driver of therapy resistance. TAMs secrete cytokines and growth factors that shield tumor cells; for example, in chordoma, tumor-derived IL-6 activates STAT3 in TAMs, polarizing them to an M2-like state and eventually enhancing tumor cell migration and invasion via TNF-α–NF-κB signaling and upregulation of matrix metalloproteinases (38). The bidirectional crosstalk between tumor cells and immunosuppressive TAMs reinforces malignancy. In GBM, the abundance of immunosuppressive TAMs contributes to poor prognosis and chemo-resistance, and the immunosuppressive TME correlates with reduced efficacy of PD-1/PD-L1 inhibitors (6). Therefore, targeted disruption of C3a/C3aR signaling to reverse immunosuppressive TAM polarization or to uncouple their malignant interactions with tumor cells holds promise as a strategy to overcome GBM invasiveness and therapeutic resistance (**Figure 1**).

## 5 Therapeutic strategies targeting C3/C3aR signaling in GBM

### 5.1 Clinical potential of C3aR small-molecule antagonists

Preclinical data support a clear rationale for C3aR antagonists in GBM therapy. The evidence summarized in this section derives primarily from preclinical models (*in vitro* cell experiments and syngeneic murine models), and no clinical trial data are currently available for C3aR antagonists in GBM patients; translational validation therefore remains an essential next step before clinical application. In GBM, C3 and C3aR are upregulated, especially in IDH-wild-type GBM and IDH-mutant grade 4 astrocytomas, with C3aR expression markedly higher than in other brain tumors (15). In syngeneic mouse models (*in vivo* preclinical), C3aR-specific antagonist SB290157, used as a single agent, significantly prolongs survival, establishing independent therapeutic value for targeting C3a/C3aR signaling (14). The fundamental mechanism is to reverse the immunosuppressive microenvironment, a critical component of which is TAMs; C3a/C3aR signaling activates Ca2+ signaling and NFAT1, promoting C3 transcription and C3a release, creating a self-reinforcing loop that sustains immunosuppressive polarization (28). Thus, C3aR antagonism can disrupt this malignant cycle, lowering immunosuppressive TAMs and shifting the TME immunosuppressive “cold” to immunostimulatory “hot”, thereby enhancing anti-tumor immunity. This provides a strong rationale for single-agent C3aR antagonism and, even more importantly, a robust foundation for combining C3aR antagonists with immune checkpoint inhibitors or other immunotherapies to improve response rates by alleviating immune suppression.

### 5.2 Synergy with radiotherapy

Combining C3aR signaling inhibitors with radiotherapy (RT) shows substantial synergistic potential (based on preclinical syngeneic murine models (14)), rooted in the shared driver of hypoxia. Hypoxia induces C3 expression, as noted in patient GBM samples and GBM models (14). Since hypoxia also drives radiotherapy resistance, targeting the hypoxia-activated C3a/C3aR axis is a rational radiosensitization strategy. Preclinical data show that SB290157 in combination with RT extends survival beyond either modality alone in tumor-bearing mice (14). The proposed mechanism: RT-induced tumor cell killing can concurrently trigger local inflammation and complement activation, creating a pro-survival and immunosuppressive feedback that limits RT efficacy; C3aR antagonism interrupts this negative feedback, reducing immunosuppressive TAM accumulation and alleviating immune suppression, thereby enhancing RT’s direct cytotoxic effects and stimulating anti-tumor T-cell responses. These findings await validation in clinical settings. This supports a “1 + 1>2” therapeutic gain from combining C3aR antagonists with RT.

### 5.3 Potential for combination with other immunotherapies

**Figure 2:**
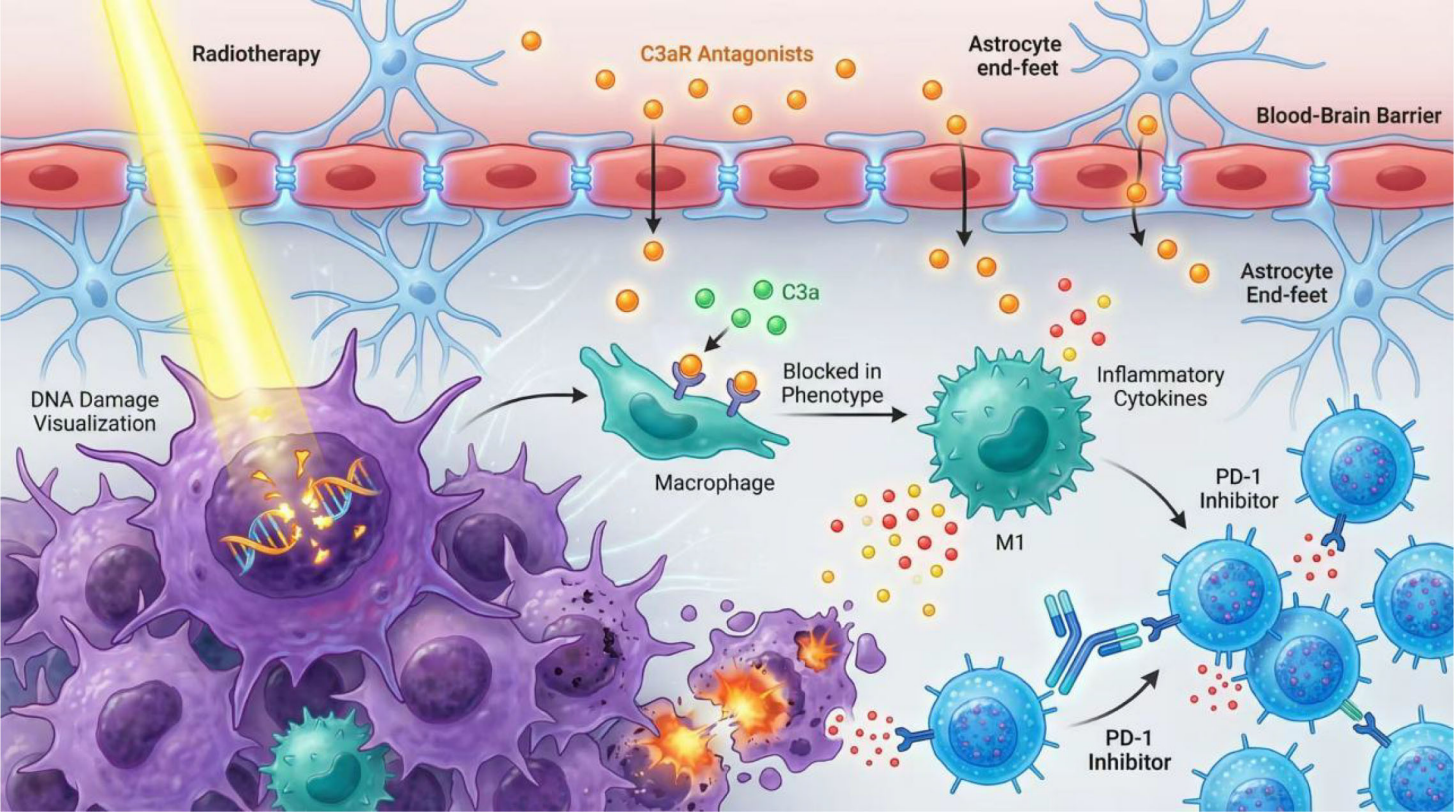
Multimodal targeting of C3aR reshapes the tumor immune microenvironment and enhances standard-of-care efficacy. A small-molecule C3aR antagonist (e.g., SB290157, based on preclinical syngeneic murine model data) penetrates the blood–brain barrier to access the tumor parenchyma, where it competitively binds C3aR on tumor-associated macrophages (TAMs), thereby interrupting C3a-driven signaling. C3aR blockade effectively reverses TAM polarization, reprogramming from pro-tumor M2-like/immunosuppressive toward anti-tumor pro-inflammatory. This shift relieves suppression of effector T cells, promotes CD8^+^ T cell infiltration and cytotoxic activity, and converts a “cold” tumor into an immunologically “hot” one. When combined with radiotherapy, C3aR antagonists can mitigate radiation-induced hypoxia-associated recruitment of M2-like/immunosuppressive TAMs, overcoming radioresistance; concomitant use with immune checkpoint inhibitors (e.g., anti-PD-1) further lifts immune suppression, substantially enhancing systemic anti-tumor immunity. All combination strategies depicted represent preclinical conceptual or early experimental rationale; no clinical trial data are currently available for C3aR antagonists in GBM patients, and translational validation remains an essential next step.

Targeting C3aR opens new dimensions for combination immunotherapy in GBM. First, there is a solid theoretical basis for pairing C3aR antagonists with immune checkpoint inhibitors (e.g., anti-PD-1/PD-L1). By reducing immunosuppressive TAMs and reversing immunosuppression, C3aR antagonism could convert “cold” tumors into more inflamed, immune-accessible tumors, thereby enhancing responsiveness to PD-1/PD-L1 blockade and improving overall efficacy (28, 39). Second, combining C3aR antagonists with TAM-targeting strategies such as CSF-1R inhibitors (which limit TAM recruitment and survival) could complementarily reprogram existing TAMs from pro-tumor immunosuppressive to anti-tumor pro-inflammatory phenotypes (28). The spatial co-localization of C3aR^+^ TAMs with VEGF-expressing cells further suggests that C3a/C3aR signaling may contribute to pro-angiogenic TAM function; although this association has not yet been causally proven, it provides a rationale for exploring combinations of C3aR antagonists with anti-angiogenic agents (e.g., bevacizumab). Such pairing may have dual effects: suppressing abnormal angiogenesis and reconditioning the immune microenvironment, thereby providing a multi-pronged assault on GBM’s malignant network. All combination strategies discussed here are currently at the preclinical conceptual or early experimental stage and require rigorous clinical evaluation. Collectively, a C3aR-centric approach can be flexibly integrated with radiotherapy, immune checkpoint blockade, TAM-targeted therapies, and anti-angiogenic treatments to address GBM’s complex biology (**Figure 2**).

## 6 C3-driven GBM liquid biopsy and biomarker development

### 6.1 C3 in plasma exosomes as a diagnostic biomarker

**Figure 3:**
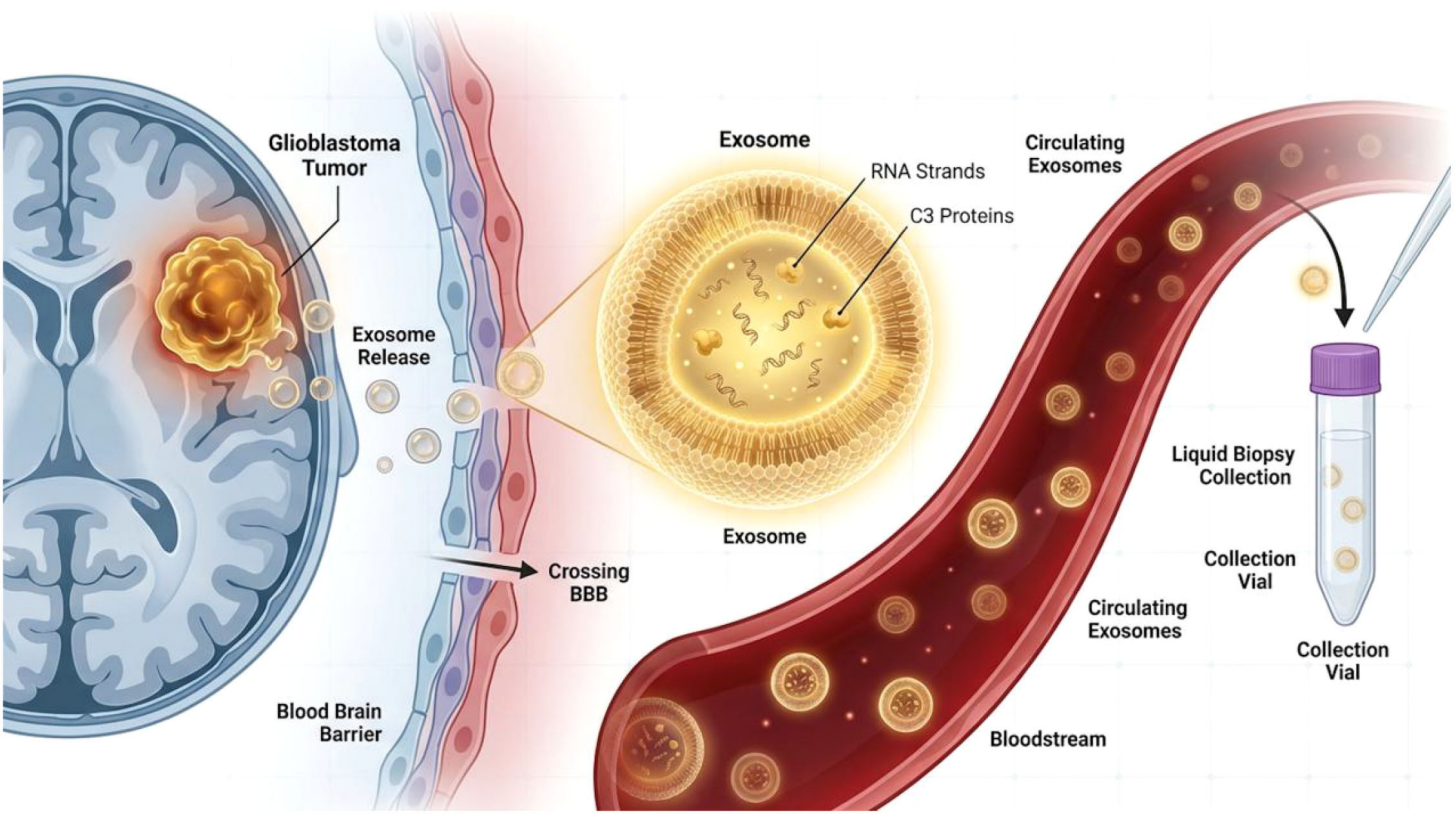
Circulating exosomal C3 as a noninvasive biomarker for glioblastoma diagnosis and dynamic monitoring. Circulating exosomal C3 as a noninvasive biomarker for glioblastoma diagnosis and dynamic monitoring. Conceptual hypothesis schematic—research hypothesis requiring experimental validation. This figure presents a research hypothesis and does not summarize established experimental findings. To date, no published studies have directly detected C3 in plasma exosomes from GBM patients or GBM models, nor established a direct association between exosomal C3 levels and GBM disease severity or treatment response. The evidence presented here is drawn from other disease contexts (pancreatic cancer, nodular vasculitis, anti-NMDA receptor encephalitis, early psychosis) as proof-of-concept only; dedicated GBM-specific experimental validation is required before clinical application can be considered. Exosomes released by glioblastoma cells and stromal components within the tumor microenvironment carry defined molecular cargo, including complement C3 protein and its related cleavage products or C3-associated transcripts. These nanoscale vesicles can cross the blood–brain barrier (BBB) or access the peripheral circulation through a compromised blood–brain tumor barrier. Based on cross-disease proof-of-concept evidence, we hypothesize that liquid biopsy-based detection of exosomal C3 levels in patient plasma may enable early adjunctive diagnosis of GBM and differential diagnosis from other intracranial lesions. Dynamic monitoring of exosomal C3 and/or C3a levels during targeted therapy (e.g., C3aR antagonists) could potentially serve as a pharmacodynamic indicator of target engagement and a surrogate for therapeutic response and recurrence risk—though these hypotheses remain to be prospectively validated in GBM-specific cohorts.

To date, no published studies have directly detected C3 in plasma exosomes from GBM patients or GBM models, nor established a direct association between exosomal C3 levels and GBM disease severity or treatment response; the evidence presented below is drawn from other disease contexts as proof-of-concept (40), and dedicated experimental validation in GBM is required before clinical application can be considered. Against this backdrop, proteomics offers powerful tools for discovering novel liquid biopsy markers (**Figure 3**). Plasma-derived small extracellular vesicles (sEVs) carry disease-relevant proteins; C3, a key inflammatory marker, has been identified as differentially expressed in exosome-rich contexts. In pancreatic cancer, PS-MIP–enriched exosomes reveal differential C3 (and VWF) levels between patients and healthy controls, identified as highly connected in protein–protein interaction networks (41). Similar mass-spectrometry–based proteomic studies in nodular vasculitis imply C3 among differential proteins in plasma and exosomes (42). These findings from non-GBM diseases demonstrate that circulating exosomes can carry C3 protein cargo and that exosomal C3 levels may reflect disease states in inflammatory conditions—providing conceptual support for investigating exosomal C3 in GBM, but not constituting direct evidence for GBM biomarker utility. In anti-NMDA receptor encephalitis, serum C3 is lower than in viral encephalitis or controls, and combined exosomal miR-140-5p with serum C3 improves diagnostic performance (AUC) (43), illustrating the feasibility of using circulating C3 (free or exosome-associated) as an auxiliary diagnostic indicator in neurological disease. Based on this cross-disease evidence, we hypothesize that assessing C3 levels in GBM patient plasma exosomes could provide a minimally invasive adjunct for diagnosis; however, given that C3 elevation is observed across many cancers and inflammatory diseases, its specificity for GBM diagnosis alone is limited. The more realistic and clinically meaningful application may lie in treatment monitoring and dynamic disease surveillance, as discussed below. Dedicated GBM-specific studies are needed to validate this hypothesis.

### 6.2 Monitoring treatment response and prognosis

Dynamic biomarker surveillance is crucial to evaluate treatment response and disease trajectory. We hypothesize that for therapies targeting the complement system, such as C3aR antagonists, tracking changes in exosomal C3 or its fragments (e.g., C3a) in body fluids before and after treatment could serve as a pharmacodynamic indicator of target engagement and a potential surrogate for therapeutic effect and recurrence risk—though this remains to be prospectively validated in GBM patients. Exosomes are known to harbor and transport activated complement fragments; for example, in certain inflammatory conditions, exosomal C3a and C5a (C5a is another bioactive complement activation fragment generated from C5 cleavage, distinct from C3a; elevated C5a has been detected in plasma in inflammatory settings, with both C3a and C5a implicated in complement-mediated inflammatory progression (44)) levels rise despite unchanged plasma C3/C5 (44). Thus, effective suppression of tumor-local complement activation might manifest as reduced exosomal C3a in circulation. Similarly, in early psychosis, astrocyte-derived exosomes (ADEs) in plasma show elevated C3b and decreased CD55 (45), hinting at neuroinflammatory state monitoring via exosomes. These findings provide proof-of-concept for exosomal complement fragments as state indicators, but direct evidence in GBM is absent. To build more precise prognostic models, integrating C3-related biomarkers with existing clinical tools is a natural progression. Imaging (MRI) provides anatomical and functional tumor information, while molecular profiling (e.g., IDH mutation status, MGMT promoter methylation) reveals tumor biology. Multimodal data integration can compensate for the shortcomings of single metrics. In hepatocellular carcinoma (HCC), a plasma exosome–based lncRNA-driven molecular typing system stratifies patients into subtypes with distinct prognosis and immune milieu (46). In metastatic castration-resistant prostate cancer (mCRPC), high plasma exosome AKR1C3 mRNA levels correlate with shorter progression-free and overall survival (47). Therefore, future work could fuse GBM patient plasma exosome C3 content, imaging features, and key genomic alterations into machine-learning–driven predictive models to assess prognosis, identify high-risk recurrence, and tailor individualized therapies—subject to prospective validation in GBM-specific cohorts.

## 7 Current challenges and future research directions

### 7.1 Delivery across the blood–brain barrier

Efficient delivery across the BBB remains a principal barrier to translating C3/C3aR-targeted therapies for GBM. The BBB protects CNS homeostasis via tight junctions and abundant efflux transporters, limiting brain penetration of most therapeutics (46). Although GBM can disrupt the BBB in tumor regions, the residual permeability is often insufficient to achieve therapeutic brain tissue concentrations (47). Therefore, developing C3aR antagonists or delivery systems capable of BBB penetration is essential. Current strategies fall into noninvasive and invasive categories. Noninvasive approaches include nanocarrier systems (lipid nanoparticles, polymeric nanoparticles, inorganic nanoparticles) that improve drug stability, exploit enhanced permeability and retention, or actively target receptors (e.g., transferrin receptor) to cross the BBB (48, 49). Focused ultrasound with microbubbles is another promising noninvasive modality that transiently and reversibly opens the BBB to markedly enhance delivery of chemotherapeutics, antibody-drug conjugates, and even large lipid nanoparticles to brain tumors (50, 51). Cell-penetrating peptides or biomimetic carriers (e.g., exosomes, red blood cell–membrane–coated nanoparticles) are also explored for BBB crossing (52, 53). However, successful translation requires not only efficient BBB penetration but also favorable intratumoral distribution and target engagement. This necessitates precise pharmacokinetics/pharmacodynamics in the brain and reliable preclinical models to predict human distribution (54). Future work should integrate deeper BBB biology, optimize delivery system design, and establish standardized evaluation frameworks to translate C3a/C3aR-targeted therapies from bench to bedside.

### 7.2 Potential systemic side effects of complement inhibition

Global inhibition of the complement system, especially the classical pathway, can raise infection risk and other systemic adverse effects, challenging safety in GBM treatment. The complement system is an integral part of innate immunity; broad classical pathway blockade can compromise host defense (55). To balance efficacy and safety, strategies should shift from “global inhibition” to “precision targeting.” This requires delineating the exact cellular sources and dominant routes of complement activation in GBM. In neuroinflammation and brain injury, reactive astrocytes and microglia contribute significantly to C3 production (56–58). In GBM, tumor cells, TAMs, and endothelial cells can contribute to localized complement activation. For example, endothelial cell surface glycosylation changes can trigger C3 activation on the luminal surface in certain brain metastases and neuropathologies, disrupting the blood–brain barrier (55). Therefore, interventions should prioritize local tumor-specific C3a/C3aR axis alterations rather than systemic complement inhibition (59). Approaches include tumor microenvironment–activated prodrugs, targeted delivery systems delivering C3aR antagonists specifically to the tumor, or site-directed complement inhibitors that suppress C3 activation in the tumor locale (60). Understanding variations of complement signaling across GBM molecular subtypes will further enable precision therapies. By focusing on tumor-local complement activation and leveraging advanced delivery technologies to constrain drug action, it may be possible to attenuate pro-tumor inflammatory signals while preserving systemic complement function and thereby widening the safety window for C3/C3aR-targeted therapies.

### 7.3 Tumor heterogeneity and resistance mechanisms

GBM’s profound intratumoral and intertumoral heterogeneity poses a major challenge to C3a/C3aR-targeted therapy and may drive adaptive resistance (61). Heterogeneity manifests in cellular morphology, transcriptomics, epigenetics, and metabolism, leading to divergent therapeutic responses within a single tumor and among patients (61). It will be essential to quantify C3a/C3aR pathway activity across molecular subtypes (e.g., classical, mesenchymal, proneural) and to delineate functional differences. MES-like GBM subtypes, which harbor higher levels of myeloid infiltration, more active inflammatory transcriptional programs, and more severe hypoxia (18), may exist in a microenvironment with elevated complement activation, and could therefore be more responsive to C3aR-targeted intervention. However, this hypothesis is inferential and requires direct experimental validation through subtype-stratified functional studies. Critically, because not all GBMs harbor equivalent C3aR^+^ TAM infiltration or C3/C3aR pathway activation, not all patients may benefit equally from C3aR antagonists. Future research should establish predictive biomarkers for patient stratification—such as baseline C3/C3aR expression levels, TAM infiltration density, and molecular subtype classification—to enable personalized treatment selection. Potential resistance mechanisms should be prospectively studied: tumor cells could circumvent therapy by upregulating alternative complement activation pathways, altering C3aR expression, or activating parallel pro-survival signaling channels (e.g., PI3K/AKT, STAT3) (62). Therapy-induced selective pressures may enrich for glioma stem cell populations with heightened self-renewal and invasive capacity, contributing to relapse. To translate hypotheses into clinical practice, advanced preclinical models are needed. Patient-derived organoids preserve the heterogeneity and architecture of primary tumors and are powerful for testing C3a/C3aR pathway function and drug sensitivity in patient-specific contexts (63). Syngeneic murine models—where immunologically intact host mice are implanted with compatible mouse glioma cells—provide an immune-competent platform to study tumor–host immune interactions and to assess the overall impact of targeted therapy on tumor growth, invasion, and survival while tracking emergent resistance. Patient-derived xenograft (PDX) models, which require immunocompromised hosts, are primarily suited for studying tumor-cell-intrinsic C3/C3aR pathway function and drug pharmacology rather than tumor–immune interactions; humanized mouse models may better recapitulate the human immune microenvironment for translational studies. Systematic studies in these complex model systems will deepen understanding of how heterogeneity influences C3a/C3aR-targeted therapy and help identify strategies to counteract potential resistance, pushing forward personalized treatment paradigms.

### 7.4 Exploration of novel therapeutic modalities

Exploring combinations of complement targeting with emerging immunotherapies opens new horizons for GBM treatment. Engineering immune cells that both block C3aR and stimulate anti-tumor immunity is a key direction. For example, chimeric antigen receptor macrophages (CAR-M) or CAR-T cells could be engineered to secrete C3aR antagonists or express membrane-bound complement regulators, thereby locally suppressing complement-mediated immunosuppression and boosting therapeutic activity and persistence (64). Bispecific antibodies that simultaneously target tumor-associated antigens and complement components (e.g., C3a) could precisely direct complement inhibition to tumor sites. Indirect strategies via epigenetic modulation are also of interest. Two independent lines of evidence support the epigenetic regulation of complement expression. First, genome-wide transcriptomic analyses in macrophages have demonstrated that HDACs are required for the expression of a broad set of host defense genes, explicitly including complement factors (65); HDAC inhibitor treatment markedly suppresses innate immune gene programs in macrophages and dendritic cells, suggesting that epigenetic state directly governs complement component expression in myeloid cells. Second, intracellular C3b has been shown to translocate to the nucleus in tumor cells and physically associate with the SIN3A/HDAC1/2 chromatin remodeling complex to epigenetically silence GADD45A, thereby conferring chemoresistance in lung cancer (66)—revealing a reciprocal relationship in which complement fragments themselves serve as epigenetic regulators. Together, these findings provide a mechanistic rationale for exploring epigenetic modifiers as indirect regulators of C3/C3aR signaling. However, direct experimental evidence that HDAC inhibitors or DNA methyltransferase inhibitors specifically regulate C3 or C3aR expression in GBM cells or GBM-associated TAMs is currently absent; this remains a scientifically well-grounded but unvalidated research direction that warrants dedicated investigation in GBM models. Co-targeting other signaling cascades that crosstalk with complement (e.g., PI3K, NF-κB, STAT3 inhibitors) could yield synergistic effects by jointly suppressing tumor-promoting inflammation while preserving anti-tumor immunity (62). Finally, nanotechnology–based multifunctional delivery systems could co-deliver complement inhibitors with immune stimulants (e.g., STING agonists, immune checkpoint inhibitors) to brain tumors, simultaneously alleviating local immunosuppression and activating robust anti-tumor immunity (67, 68). These innovative therapeutic modalities aim to overcome the limitations of single-agent therapy through multi-target, multi-mechanism synergy to achieve more effective and durable GBM control.

## 8 Conclusion

The C3/C3aR signaling axis occupies a central hub in the GBM tumor microenvironment, orchestrated by hypoxia and TGF-β1 among other drivers. It not only promotes malignant tumor behavior directly but also links hypoxia, immunosuppression (notably immunosuppressive/M2-like TAM polarization), and aberrant angiogenesis (via VEGF upregulation, though the causal link requires further functional validation) into a cohesive pro-tumor network. Importantly, the available evidence supporting the pro-tumorigenic roles of C3/C3aR and the therapeutic potential of C3aR antagonists derives predominantly from preclinical models (*in vitro* studies and syngeneic murine models); clinical evidence is lacking and translational validation is required. From a translational perspective, high C3 and C3aR expression correlate with poor prognosis, lending themselves to potential liquid-biopsy–based diagnostics and prognostication—though the diagnostic specificity of C3 alone is limited, and its role in treatment monitoring and dynamic surveillance may be more realistic. Preclinical data provide compelling evidence for the therapeutic potential of targeting C3aR with small-molecule antagonists, alone or in combination with radiotherapy or other immunotherapies, to reverse immunosuppressive TMEs and prolong survival in GBM models. While promising, translating this vision to the clinic requires overcoming several challenges: delivering therapeutics across the BBB, ensuring target specificity and safety by limiting systemic complement inhibition, addressing GBM’s pronounced heterogeneity and adaptive resistance, and identifying predictive biomarkers to guide patient selection and monitor response. Given intertumoral heterogeneity, not all GBM patients may harbor sufficient C3aR^+^ TAM infiltration to benefit from C3aR antagonism; subtype stratification and predictive biomarker development are therefore integral to a precision medicine approach. Future research should focus on delivery optimization, precision targeting of tumor-local complement activation, validation in patient-derived models and clinical trials, and exploration of synergistic combinations with radiotherapy, immune checkpoint blockade, TAM-targeted therapies, and anti-angiogenic strategies. Together, targeting the C3/C3aR axis represents a highly promising new avenue with the potential to disrupt the malignant TME at its roots and to improve outcomes for patients with GBM.
